# RORα and 25-Hydroxycholesterol Crosstalk Regulates Lipid Droplet Homeostasis in Macrophages

**DOI:** 10.1371/journal.pone.0147179

**Published:** 2016-01-26

**Authors:** Zewen Kelvin Tuong, Patrick Lau, Ximing Du, Nicholas D. Condon, Joel M. Goode, Tae Gyu Oh, Jeremy C. Yeo, George E. O. Muscat, Jennifer L. Stow

**Affiliations:** 1 Institute for Molecular Bioscience, The University of Queensland, Brisbane QLD 4072, Australia; 2 School of Biotechnology and Biomolecular Sciences, The University of New South Wales, Sydney NSW 2052, Australia; Fundação Oswaldo Cruz, BRAZIL

## Abstract

Nuclear hormone receptors have important roles in the regulation of metabolic and inflammatory pathways. The retinoid-related orphan receptor alpha (Rorα)-deficient *staggerer* (*sg/sg)* mice display several phenotypes indicative of aberrant lipid metabolism, including dyslipidemia, and increased susceptibility to atherosclerosis. In this study we demonstrate that macrophages from *sg/sg* mice have increased ability to accumulate lipids and accordingly exhibit larger lipid droplets (LD). We have previously shown that BMMs from *sg/sg* mice have significantly decreased expression of cholesterol 25-hydroxylase (Ch25h) mRNA, the enzyme that produces the oxysterol, 25-hydroxycholesterol (25HC), and now confirm this at the protein level. 25HC functions as an inverse agonist for RORα. siRNA knockdown of Ch25h in macrophages up-regulates *Vldlr* mRNA expression and causes increased accumulation of LDs. Treatment with physiological concentrations of 25HC in *sg/sg* macrophages restored lipid accumulation back to normal levels. Thus, 25HC and RORα signify a new pathway involved in the regulation of lipid homeostasis in macrophages, potentially via increased uptake of lipid which is suggested by mRNA expression changes in *Vldlr* and other related genes.

## Introduction

Lipids are essential for almost all aspects of eukaryotic life but, in excess, lipids are cytotoxic. In cells, lipids are safely stored in lipid droplets (LDs)—monolayer membrane-encased organelles which are managed as dynamic repositories of lipids (mainly in the form of cholesterol esters and triglycerides) [1, 2]. LDs also represent locations where key enzymes involved in cholesterol metabolism and fatty acid synthesis regulate the various anabolic and catabolic steps in lipid metabolism [3, 4]. Macrophages are innate immune sentinel cells that also play important roles in the regulation of lipid homeostasis. Complex pathways in these cells regulate lipid uptake, synthesis, storage, and efflux, with LD status reflecting the balance of these processes [1, 2]. Lipid metabolites can induce chronic inflammation by promoting macrophage infiltration and activation within tissues [5–7]. The damaging effects of cellular lipid overload are well documented but not fully understood in atherosclerosis and certain metabolic disorders coupled to insulin resistance [8]. Macrophage LD biogenesis represents an important focal point in the understanding of atherosclerosis development [9].

Nuclear hormone receptors operate in transcriptional regulation at the junction of metabolism and inflammation. One focus of current research on obesity and diabetes is on the retinoid-related orphan receptor alpha (RORα) and its roles in glucose and lipid metabolism. *In vitro* studies on RORα coupled with *in vivo* data derived from the Rorα-deficient *staggerer* (*sg/sg*) mice highlight roles for this receptor in regulating lipid homeostasis and metabolism [10, 11]. Despite being congenitally lean, *sg/sg* mice have dysregulated inflammation, innate immunity and develop more severe atherosclerosis [12, 13]. However, the link between RORα and lipid metabolism is not fully understood.

Oxysterols, including 25-hydroxycholesterol (25HC), are emerging as key coordinators of metabolic and inflammatory processes [14, 15]. 25HC is produced by the enzyme cholesterol 25-hydroxylase (Ch25h) and belongs to a family of bioactive cholesterol derivatives produced by cells in response to fluctuating cholesterol levels and also during infection [14]. Specifically, 25HC is known to be a potent suppressor of cholesterol biosynthesis and is involved in the up-regulation of cholesterol esterification [16, 17]. 25HC functions as an agonist for liver X receptor alpha (LXRα) and it is an inverse agonist for RORα [18–22]. We previously showed that bone marrow-derived macrophages (BMMs) from *sg/sg* mice displayed significantly reduced mRNA expression of Ch25h and deficient phagocytosis [23]. Although transient expression of RORα in macrophages induced Ch25h mRNA expression [23], the transcriptional regulation of Ch25h by RORα remains elusive. As the range and scope of Ch25h and 25HC continues to develop, it is clear that crosstalk between NR activity and oxysterol (25HC) signaling represents an important nexus in macrophage biology and the regulation of cholesterol homeostasis and inflammation.

Here we examined BMMs from *sg/sg* mice and found that lipid storage is altered in these cells and the findings we present herein reveal a new pathway for regulating lipid homeostasis and cholesterol trafficking in macrophages, mediated jointly through RORα and 25HC.

## Experimental Procedures

### Animals

16 week old male wild-type (WT) mice and *sg/sg* littermates were obtained from crossing heterozygous *sg/+*, B6.C3(Cg)-*Rorα*^*sg*^/J. Animals were housed in the Queensland Bioscience Precinct Vivarium (UQ) with a 12 h light-dark cycle and feeding has been previously described [10]. Mice were fasted for 6 h and euthanized by CO_2_ asphyxiation. All aspects of animal experimentation were approved by The University of Queensland Animal Ethics Committee.

### Bone marrow-derived macrophages

BMMs were obtained by *ex vivo* differentiation of bone marrow cells isolated from femur and tibia [24]. BMMs were differentiated for 7 days in humidified 5% CO_2_ at 37°C in complete medium (Roswell Park Memorial Institute (RPMI)-1640 (BioWhittaker, Lonza, VIC, Australia), supplemented with 10% fetal calf serum (Thermo Fisher Scientific, VIC, Australia), 1% L-glutamine (Invitrogen, Life Technologies, VIC, Australia), 20 U/mL penicillin (Sigma-Aldrich, NSW, Australia), 20 μg/mL streptomycin (Invitrogen), and 100 ng/mL purified recombinant macrophage-colony-stimulating-factor-1 (M-CSF-1) (Bio-Rad Laboratories, NSW, Australia) [24].

### Treatments

25HC (Sigma-Aldrich) was dissolved in DMSO and treatments were carried out at varying concentrations (final DMSO concentration 0.01%). Oleic acid (Calbiochem, San Diego, CA, USA) was prepared as per [25] and used at a final concentration of 400 μM in the form of fatty acid-supplemented medium for 4 h. Acetylated-LDL (acLDL) experiments were conducted using fluorescently-conjugated Alexa Fluor^®^ 594 acLDL acquired from Molecular Probes.

### Lipid extraction for thin layer chromatography (TLC)

Cells were seeded on 100 mm dishes at 85% confluency and allowed to grow until harvesting. Cells were thoroughly rinsed with PBS and lysed using 2 mL 0.1 M NaOH. Lysates were then transferred to glass screw-capped or 10 mL falcon tube and dishes were washed with 1800 μL PBS and 200 μL 1 M HCl. Cell lysates were then pooled and tap-mixed to get homogenous solution. Protein concentration was determined using BCA protein assay kit (Pierce). Samples were normalized to the same protein concentration and then 2 mL methanol was added to the tubes followed by 2 mL Hexane. Tubes were vortexed and centrifuged for 5 min at 1000 g. The hexane layer (top) was transferred to a 2 mL eppendorf tube. Tubes were allowed to air dry overnight in the fume hood. Lipids were then resuspended in 60 μl hexane and spotted on a silica gel G TLC plate, which was developed in the solvent system hexane/diethyl ether/acetic acid (85:15:1). Once the lipids were separated, the TLC plates were stained by iodine vapour and scanned.

### acLDL uptake assay

BMMs were seeded overnight onto glass coverslips prior to acLDL uptake assay. Alexa Fluor^®^ 594 acLDL was prepared in complete RPMI media containing 10% fetal calf serum and 1% L-glutamine at 1:200 dilution (final acLDL concentration 5 μg/mL). Coverslips were inverted onto 25 μL of acLDL for 10 min at 4°C to facilitate binding to surface receptors. Cells were then incubated at 37°C for 1 h to allow for acLDL uptake and subsequently fixed using 4% paraformaldehyde (PFA)/PBS.

### Fixed-cell fluorescence imaging

Fixed-cell fluorescent images were captured using an Olympus BX-51 (Olympus Imaging Systems, PA, USA) upright epifluorescence microscope equipped with Plan Apo oil immersion objectives (40x: numerical aperture (n.a.) 1.00 oil; 60x: n.a. 1.35). Images were captured with an Olympus DP-71 12 megapixel color camera using the Olympus DP controller version 2.1 capture software. Images requiring z-series acquisition were acquired using a Personal DeltaVision Olympus IX71 (Olympus) inverted wide-field deconvolution microscope equipped with U-Apochromat 40x (n.a. 1.35) and Plan-Apochromat 60x (n.a. 1.42) oil immersion objectives. Images were typically sequentially captured at 0.1 μm step-intervals (30-slices) using a 12 megapixel Roper Coolsnap HQ2 monochrome camera and softWoRx^®^ image acquisition software. In all cases, images were cropped and optimized using Adobe Photoshop CS6 (Adobe Systems Incorporated, San Jose, USA). All image analyses were performed using ImageJ software (version 1.47m and later) (NIH, USA).

### Lipid droplet staining

All cells were fixed using 4% PFA/PBS for 1 h, washed and stored in PBS at 4°C prior to staining. LDs were labeled using either Oil Red O (Sigma-Aldrich, NSW, Australia) or anti-adipophilin (ADRP) (N-terminal, guinea pig polyclonal GP40) antibody in fixed cells. Briefly, Oil Red O powder was dissolved in 100% isopropanol and filtered twice using filter paper to achieve a stock solution. To make up the working solution, the stock Oil Red O solution was mixed with deionized water in a 3:2 mixture (final isopropanol concentration 60%) and filtered twice again before adding onto cover slips. The staining was performed at 4°C for 1 h and cover slips were washed with copious amounts of PBS for 10 min to remove excess Oil Red O not within LDs. Alexa Fluor^®^ 488 wheat germ agglutinin (Molecular Probes, 1:200) and DAPI (1:1000) were then added to cover slips for 30 min to label for cell outline and nucleus, respectively.

ADRP staining was performed on cells permeabilized for 10 min with 0.5% saponin/PBS. Briefly, cover slips were blocked with the blocking buffer (0.5% bovine serum albumin/PBS) for 30 min and subsequently incubated with guinea pig primary antibodies against ADRP (Progen Biotechnik, Heidelberg, Germany) for 1 h at room temperature. Coverslips were then washed with the blocking buffer for 30 min (twice) and incubated with the secondary antibody − Alexa Fluor^®^ 555 goat anti-guinea pig IgG (Molecular Probes, Invitrogen, Life Technologies, VIC, Australia). 0.5% bovine serum albumin/PBS was used as the blocking buffer and antibody dilutions for all steps. Alexa Fluor^®^ 488 Phalloidin (1:1000, Molecular Probes) and DAPI (1:1000, Molecular Probes) were used to label for cell outline and nucleus respectively during the secondary antibody incubation step.

All cover slips were mounted onto glass slides using Prolong Gold Anti-fade (Molecular Probes) and stored at 4°C until imaging.

### Lipid droplet analysis

For LD quantification, cell outline (wheat germ agglutinin or phalloidin) was masked and applied to LDs (Oil Red O or ADRP) to mask only droplets within cells. In both cases, resulting LDs in the Oil Red O or ADRP channels were converted to binary for measurement ImageJ software (NIH, USA). All structures within Oil Red O images from 100–500 cells in random fields of view were analyzed for fluorescence intensity (using integrated density) (n = 4 biological replicates for i) WT versus *sg/sg* littermates, and ii) control siRNA versus Ch25h siRNA). For ADRP images, individual LDs were selected for analysis if they can be individually distinguished from another LD with a size of >0.05 μm^2^ and a circularity index of 0.8–1. A total of i) 700–1300 LDs (WT versus *sg/sg*; n = 4 littermate pairs) and ii) 500–700 LDs (control siRNA versus Ch25h siRNA; n = 4 biological replicates) from 10 random fields of view per animal (with at least one cell displaying LDs) in each instance was analyzed.

### Electron microscopy

Cells for electron microscopy were treated with 400 μM oleic acid (Calbiochem) overnight and then fixed with 2.5% glutaraldehyde, post-fixed with 1% osmium tetroxide, and *en bloc* stained with 2% uranyl acetate. The cells were then dehydrated through a series of ethanols before embedding in LX112 resin (all from ProSciTech, Thuringowa, Queensland, Australia). Thin sections were obtained using a UC6 ultramicrotome (Leica) before viewing on a Jeol 1011 transmission electron microscope (JEOL Australasia Pty Ltd). Images were captured using iTEM software (Soft Imaging System, Olympus). Quantification of LD size was performed using ImageJ.

### Western Blot analysis

For detection of Ch25h protein, the monoclonal mouse anti-Ch25h antibody from the 18G9 hybridoma cell line was generously gifted by David Russell (UT Southwestern Medical School, USA) and blotting protocol was strictly performed as described in [26]. Briefly, microsomal membrane fractions were prepared from BMMs by ultracentrifugation [27]. Protein concentration of membrane fraction was measured using BCA protein assay (Thermo Fisher Scientific). 25 μg aliquots were solubilized in buffers described in [26] and separated on 12% sodium dodecyl sulfate-polyacrylamide gel electrophoresis (SDS-PAGE) gels for 1–1.5 h at 100–150 V. Rabbit anti-endoplasmic reticulum protein 72 (ERp72) (D70D12) antibody was acquired from Cell Signaling Technology (Genesearch, QLD, Australia) and was used at 1:1000 dilution.

Precision Plus Protein^™^ prestained protein ladders (Bio-Rad Laboratories, NSW, Australia) were used to track protein separation. Proteins were transferred to Immobilon^™^-P (Millipore, Massachusetts, USA) polyvinyldene fluoride (PVDF) membranes using the a semi-dry transfer system for 1.5 h at 180 A. Tris-buffered solution [150 mM NaCl, 10 mM Tris-HCl pH 7.5, 0.1% Tween-20] (TBS-T) containing 5% skimmed milk was used as the blocking buffer and antibody diluent in all cases. Membranes were blocked in the blocking buffer prior to incubation with primary antibody overnight at 4°C. Membranes were then thoroughly washed with TBS-T before incubation with horseradish peroxidase (HRP)-conjugated anti-mouse antibodies [1:5000 dilution, acquired from Zymed (Life Technologies, VIC, Australia)] for 1 h at room temperature. Following repeated washes with TBS-T, HRP signals were detected via enhanced chemiluminescence (ECL) using the SuperSignal West Pico Chemiluminescent Substrate Kit (Pierce, Thermo Fisher Scientific, Rockford, Illinosis, USA) as per manufacturer’s instructions. Bands were scanned and quantitated using the ImageJ gel analysis function as described in the ImageJ documentation (NIH, USA).

### siRNA transfection

siRNA transfection to knockdown expression of Ch25h is as described in [23]. Briefly, Stealth^™^ siRNA oligomers (Invitrogen) were used to knockdown expression of target gene: Ch25h (AGCCAGAUGUUAAUCACGUGAAAGG and AUCAGGACGUGACUUAGGAGCUGGA). Silencer^®^ Select Negative Control No. 1 siRNA (Ambion, Life Technologies, VIC, Australia) was used as the control siRNA for comparison. Macrophages were plated at 50% confluency on 24-well and 6-well plates and were typically transfected with 20 nM (final) of siRNA over 24 h. Transient transfection into macrophages was performed using the Lipofectamine^™^ RNAiMAX system (Invitrogen) for lipid-based delivery into cells. siRNA was combined with Lipofectamine^™^ RNAiMAX in Opti-MEM serum-free media and incubated at room temperature for 20 min, before incubating with cells in complete medium for 24 h at 37°C. The degree of mRNA knockdown was assessed by qPCR.

### Plasmids and transfection

The C-terminus HA-tagged full length human VLDLR construct in a modified pcDNA3.1 vector was generously gifted by Anna Calkin (Baker IDI, Australia). Transient transfection into RAW264.7 macrophages was performed using the Lipofectamine^™^ 2000 system (Invitrogen) for lipid-based delivery into cells. Macrophages at about 40–50% confluence on 24-well plates with cover slips were typically transfected with 2 μg of DNA combined with Lipofectamine^™^ 2000 in Opti-MEM serum-free media and incubated at room temperature for 20 min, before incubating with cells in Opti-MEM for 2 h at 37°C. The media was then replaced with RPMI-1640 complete medium and allowed to recover. Cells were subsequently fixed at the 48 h time point and stained for LDs.

### Cell lines

HA-VLDLR transfection experiments utilized cells from the RAW264.7 murine macrophage cell line (ATCC, Rockville, MD, USA) which were grown in RPMI-1640 (complete) medium supplemented with 10% heat-inactivated fetal calf serum and 1% L-glutamine in humidified 5% CO_2_ at 37°C [28]. Cells were plated at required densities onto tissue culture dishes (Corning Costar, Cambridge, MA, USA) with 12 mm glass cover slips.

### RNA extraction and purification and cDNA synthesis

Total RNA was extracted from BMMs using TRI-Reagent (Sigma-Aldrich, St. Louis, MO) and RNA purification was performed using the RNeasy mini kit (Qiagen, Clifton Hill, Victoria, Australia) according to the manufacturers’ instructions. Complementary DNA (cDNA) was synthesized from 0.5–1 μg of purified total RNA using Superscript III Reverse Transcriptase (Invitrogen) and random hexameric primers according to the manufacturer's instructions.

### qPCR analysis

Relative expression of genes was determined using the Applied Biosystems (ABI) ViiA^™^ 7 Real-Time PCR System (ABI, Singapore) as previously described [10, 23]. Relative gene expression was analyzed by qPCR using either TaqMan PCR master mix and TaqMan Gene Expression Assays (ABI, Foster City, CA), SYBR green master mix and *Mus musculus* primer sequences designed for SYBR assays, or 96-well RT^2^ Profiler PCR Array (Lipoprotein Signaling and Cholesterol Metabolism PAMM-080Z) containing RT^2^ SYBR Green Mastermix (Qiagen). Standard qPCR cycles was performed in all cases; polymerase activation step at 95°C for 10 min, followed by 45 cycles of two-step thermal cyclic PCR at 95°C for 15 sec and 60°C for 1 min. TaqMan primers for *Vldlr* (Mm00443298_m1) and *Hprt1* (Mm00446968_m1) were used.

### qPCR data analysis using StatMiner

Data from the Qiagen PCR array was analyzed using the StatMiner version 4.1 (ABI/Integromics) software package, and significance was assigned by applying empirical Bayes statistics [29]. Briefly, raw data from the Lipoprotein Signaling and Cholesterol Metabolism PAMM-080Z (Qiagen) array was uploaded into the StatMiner software package for relative quantification analysis of differentially expressed genes using comparative Ct method and statistical analysis. The Genorm software embedded within the ABI/integromics StatMiner V4.1 software package was used to compute least expression variation and select the most appropriate, stable & robust internal control genes with which to normalize the expression data against the median of the most stable endogenous controls (assays for *Actb*, *B2m*, *Gapdh*, *Gusb*, and *Hsp90ab1* were available on the array). *Actb*, *Gapdh* and *Hsp90ab* were determined to be the most stable endogenous controls. Differentially expressed genes were identified by Linear models (contained in the LIMMA package for Bioconductor R embedded in StatMiner) and significance was assigned by the application of the empirical Bayes statistic when P<0.05. While standard error bars are not included in the package, the analysis returns the empirical Bayes log odds of differential expression (B-value/score), (i.e. the probability that a gene is differentially expressed). A higher B-score represents a more significant result. Analysis also includes a *t*-score, the empirical Bayes moderated *t*-statistic (a variant *t*-test), an empirically moderated estimate of standard error/deviation from the mean. Relative quantification (RQ) is expressed as the calculated fold-differences between target genes in Ch25h knockdown BMMs and reference samples (negative control siRNA BMMs).

### Statistical analyses

Unless otherwise stated, all statistical analyses were performed using Graphpad Prism version 5.0 (Graphpad Software, San Diego, USA). Significance was calculated using unpaired two-tailed Student’s *t*-tests, one-way ANOVAs with Bonferroni’s post-tests, and two-way ANOVAs with Bonferroni’s post-tests where applicable.

## Results

### Accumulation of lipid droplets in *sg/sg* BMMs

**Fig 1:**
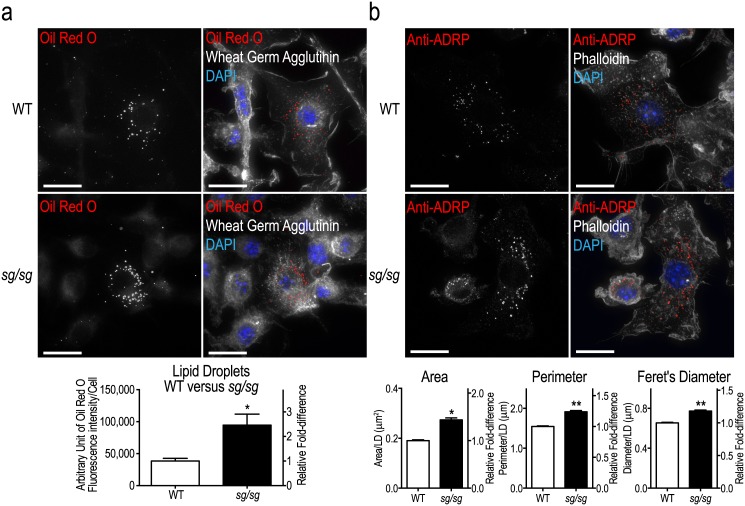
Assessment of lipid storage in WT and *sg/sg* BMMs. (a) Representative images of BMMs from WT and *sg/sg* littermates fixed and stained for LDs using Oil Red O (red). Images were taken using the Personal Deltavision Deconvolution microscope with wheat germ agglutinin (white) and nucleus (DAPI; blue) labeling. Quantification of LDs is expressed as the mean ± S.E.M. fluorescence intensity (integrated density) of stained LDs normalized to number of cells analyzed (n = ~100 cells/mouse) from n = 4 littermate pairs. (b) Representative images of WT and *sg/sg* BMMs immunolabeled for ADRP (red). Images were taken using the Personal Deltavision Deconvolution microscope (maximum projection of 30-step 0.1 μm slices) with actin (white) and nucleus (DAPI; blue) labeling. Quantification of LDs was performed on 700–1300 LDs acquired from 10 fields of view per biological replicate (n = 3 littermate pairs) and is expressed as the mean ± S.E.M. top-down cross sectional area, perimeter, and Feret’s diameter (distance between two parallel tangential planes) per LD. Statistical analyses for were performed using unpaired two-tailed Student’s t-tests where *P<0.05; **P<0.01. Scale bars = 10 μm.

Lipid absorption and efflux is an active process in macrophages and intermediate storage of lipids occurs in lipid droplets (LDs). The *sg/sg* mouse phenotype is associated with aberrant lipid handling (resistance to diet-induced obesity and hepatic steatosis, low serum triglycerides and total/HDL cholesterol) [10, 13] but lipid handling in *sg/sg* macrophages had not been assessed. We used the fluorescent lipid dye Oil Red O to stain wild-type (WT) and *sg/sg* BMMs. Discrete LDs were seen scattered through the cytoplasm (Fig 1a), but *sg/sg* BMMs appeared to have more LD staining. This was confirmed by quantification of the overall fluorescence intensities in cells which showed a ~2.5-fold increase in *sg/sg* LDs compared to WT BMMs (Fig 1a).

To confirm this finding with another LD marker, we immunolabeled adipophilin (ADRP), a well-known LD marker that is part of the perilipin family [30]. In both WT and *sg/sg* BMMs, ADRP staining was localized to the perinuclear region of cells as punctate spots or discrete ring-like structures consistent with Oil Red O-labeled LDs (Fig 1b). Using confocal imaging, we found that on average, individual LDs in WT cells had an area of ~0.2 μm^2^, perimeter of ~1.5 μm, and diameter of ~0.6 μm (Fig 1b). In contrast, we found that LDs in *sg/sg* cells were significantly larger (area increased by ~1.5-fold, perimeter by ~1.25-fold, and diameter by ~1.2-fold) (Fig 1b). Thus both labeling approaches show that *sg/sg* BMMs have an increased tendency to accumulate LDs of increased size, which is consistent with the overall lipid dysregulation in this mouse model.

**Fig 2:**
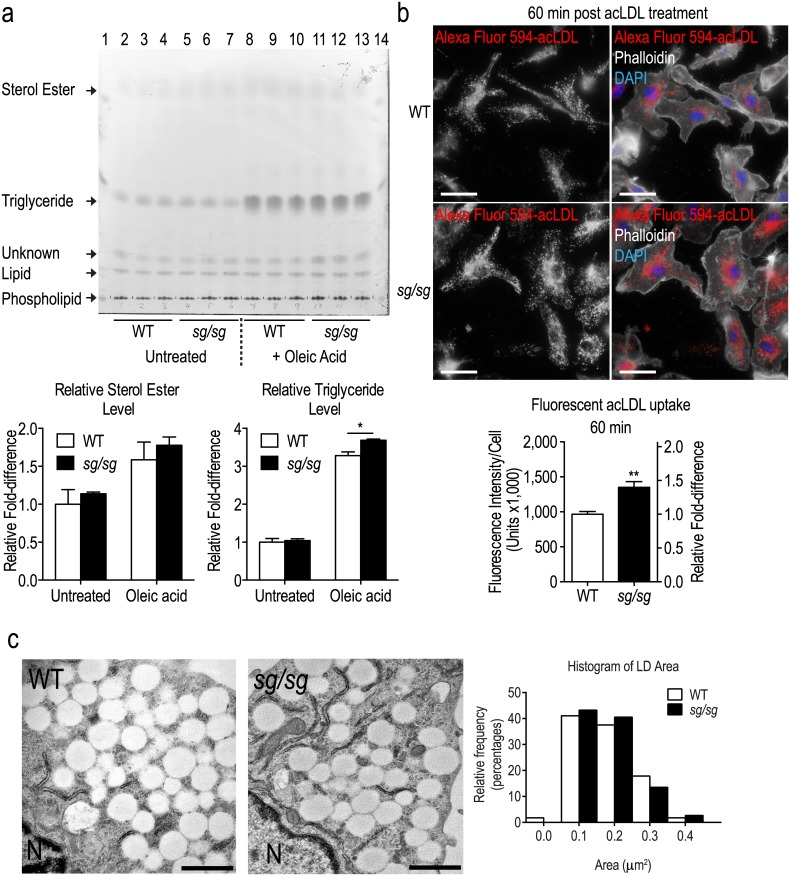
Assessment of lipid uptake in WT and *sg/sg* BMMs. (a) TLC was performed on lipid extracted from WT and *sg/sg* BMMs (n = 3 littermate pairs). Lanes 2–4 and 5–7 correspond to untreated WT and *sg/sg* samples respectively while lanes 8–10 and 11–13 correspond to 4 h 400 μM oleic acid-treated WT and *sg/sg* samples respectively. Lanes 1 and 14 are ladder standards. Quantification was performed by normalizing the relative density of triglyceride bands in each lane to WT untreated controls (arbitrarily set as 1.0) and is expressed as the mean ± S.E.M. relative fold-difference (n = 3 littermate pairs). Statistical analyses were performed using two-way ANOVAs with Bonferroni post test applied where *P<0.05. (b) Representative images of WT and *sg/sg* BMMs with endocytosed fluorescent-acLDL were captured using the Olympus upright wide-field epifluorescence microscope with actin (phalloidin; white) and nucleus (DAPI; blue) labeling, and fluorescent-acLDL (red). Quantification of fluorescent-acLDL uptake is expressed as the mean ± S.E.M. fluorescence intensity (integrated density) normalized to number of cells analyzed (n = ~200 cells per mouse) from n = 4 biological replicates (littermate pairs). Relative fold-difference is calculated with the mean of WT values set as 1.0 and is available on the right Y-axis. Statistical analysis was performed using an unpaired Student’s t-test where **P<0.01. Scale bars = 20 μm. (c) Electron microscopy of LDs. Representative images of WT and *sg/sg* BMMs treated with 400 μM oleic acid overnight show clusters of LDs accumulated in the cytoplasm. And interspersed with endoplasmic reticulum. N = nucleus, scale bars = 1 μm. Quantification of LD size is expressed as a histogram of area/LD frequency distribution.

We assessed the capacity of *sg/sg* BMMs to store fatty acid and cholesterol. Similar levels of sterol ester and triglyceride were found in WT and *sg/sg* BMMs (Fig 2a). Treatment with oleic acid (monounsaturated fatty acid) increased sterol ester and triglyceride accumulation in both WT and *sg/sg* BMMs, with a modest but significant increase (~15%) in triglyceride levels in the *sg/sg* BMMs (Fig 2a). There were no significant differences in sterol ester levels between WT and *sg/sg* BMMs under both conditions. Secondly, we assessed how well *sg/sg* macrophages take up fluorescently-tagged acetylated LDL (acLDL) and tracked the internalization of the probe. These particles hold variable amounts of cholesterol, triglycerides and phospholipids and macrophages readily take up these particles via the scavenger receptor pathway. Both WT and *sg/sg* BMMs internalized acLDL and, interestingly, there was a ~1.4-fold increase in accumulation of the probe in *sg/sg* BMMs (Fig 2b). WT and *sg/sg* BMMs were incubated with oleic acid and then prepared for electron microscopy to view their LDs at an ultrastructural level (Fig 2c). BMMs from both sources contained clusters of translucent, lipid filled, single-membrane LDs. Quantification showed no difference in the size of oleic-acid enhanced LDs in WT or *sg/sg* cells indicating that both can maximally accumulate lipid and there are no inherent morphological changes in *sg/sg* LDs. Thus normally, *sg/sg* BMMs display increased lipid absorption and storage, consistent with the increased LD size and lipid accumulation in these macrophages.

### 25-hydroxycholesterol regulates lipid droplets in macrophages

The oxysterol-converting enzyme Ch25h is expressed exclusively in immune cells, such as macrophages and dendritic cells, and it converts cholesterol into 25HC. 25HC can manipulate cholesterol levels that are stored or synthesized and therefore dictate lipid homeostasis in cells [16, 17]. We previously reported that *sg/sg* BMMs have reduced expression of Ch25h at the mRNA level [23] which affected their ability to perform the cholesterol-dependent process of phagocytosis. Here we explored a possible role for Ch25h in LD homeostasis in *sg/sg* macrophages.

**Fig 3:**
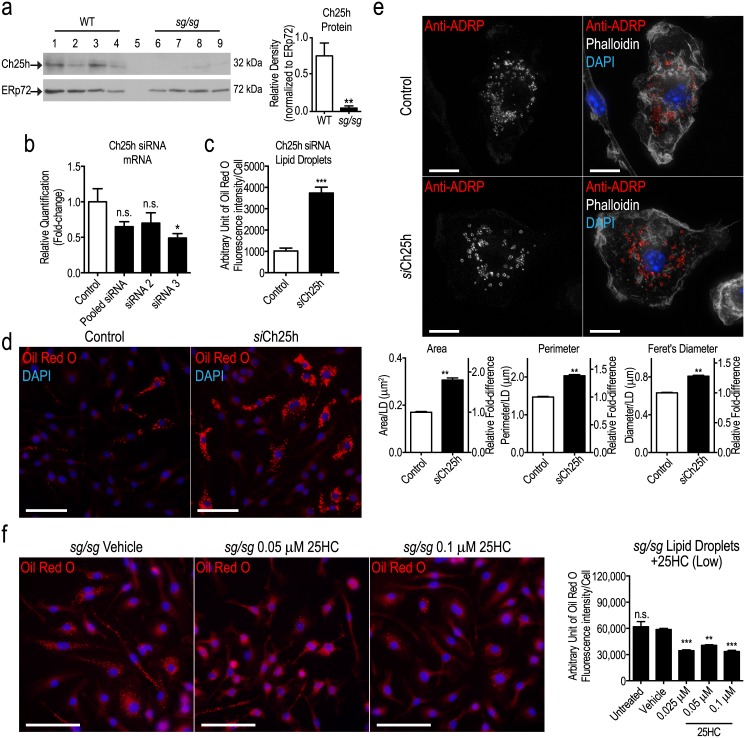
*Ch25h* knockdown up-regulates LDs. (a) Western blot analysis of Ch25h protein in WT (lanes 1 to 4) and *sg/sg* (lanes 6 to 9) BMMs (n = 4 littermate pairs). Lane 5 was only loaded with sample buffer. Quantification was performed by normalizing the relative densities of Ch25h bands (32 kDa) to the loading control ERp72 (72 kDa) and is represented as the mean ± S.E.M. relative density from n = 4 littermate pairs. Statistical analysis was performed using an unpaired two-tailed Student’s t-test where **P<0.01. (b) Two separate siRNA oligomers (individually or pooled) were used to knockdown expression of *Ch25h* in WT BMMs. The degree of mRNA knockdown was assessed by qPCR in unactivated macrophages with *Hprt1* as the housekeeping gene and represented as the mean ± S.E.M relative quantification (fold-change) with WT cells treated with siRNA negative control set as 1.0. Statistical analysis was performed using a one-way ANOVA with Bonferroni’s post test applied, comparing Ch25h mRNA expression in all treatments relative to the siRNA negative control (n = 4 biological replicates) where *P<0.01; n.s. denotes non-significant. (c) Quantification of LDs in WT control and *si*Ch25h BMMs is expressed as the mean ± S.E.M. fluorescence intensity (integrated density) of stained LDs (Oil Red O) normalized to number of cells analyzed (~500–700 cells per biological replicate (n = 4)). Statistical analysis was performed using an unpaired two-tailed Student’s t-test where ***P<0.001. (d) Representative images of LDs (stained using Oil Red O) in WT control and *si*Ch25h BMMs. Images were taken using the Olympus upright wide-field epifluorescence microscope. Blue denotes nucleus (DAPI) labeling and red denotes LD (Oil Red O) labeling. Scale bars = 50 μm. (e) Representative images of anti-ADRP (red) labeling in WT control and *si*Ch25h BMMs. Images were taken using the Personal Deltavision Deconvolution microscope and displayed as the maximum projection of 30-step z-stack (0.1 μm slices). White denotes actin (phalloidin) labeling and blue denotes nucleus (DAPI) labeling. Quantification of LDs was performed on 600–700 LDs acquired from 5 fields of view per biological replicate (n = 4 biological replicates) and is expressed as the mean ± S.E.M. cross sectional area, perimeter, and Feret’s diameter per LD. Statistical analyses were performed using unpaired two-tailed Student’s t-tests for each parameter where **P<0.01. Scale bars = 10 μm. (f) *sg/sg* BMMs treated with vehicle control (0.01% DMSO) or 25HC (0.025 μM to 0.1 μM) for 4 h. Representative images were taken using the Olympus upright wide-field epifluorescence microscope are shown on the left panel where blue denotes nucleus (DAPI) labeling and red denotes LD (Oil Red O) labeling. Quantification of LDs (low 25HC treatment) is expressed as the mean ± S.E.M. fluorescence intensity (integrated density) of stained LDs normalized to number of cells analyzed (n = ~100 cells per mouse per treatment). Statistical analysis was performed using a one-way ANOVA with Bonferroni’s post test (n = 3 biological replicates), comparing the amount of LDs in each treatment to the vehicle control *sg/sg* cells where **P<0.01; ***P<0.001; n.s. denotes non-significant. Scale bars = 50 μm.

First, we confirm that Ch25h is indeed attenuated in the *sg/sg* model by showing that, at the protein level, the level of this enzyme is dramatically reduced (~90%) in *sg/sg* BMMs (Fig 3a). Thus the increase in LDs in *sg/sg* BMMs coincides with the near absence of Ch25h. If the two processes are associated, we would predict that reducing Ch25h in WT cells should change LD status.

We employed specific siRNAs targeting *Ch25h* [23] to knockdown the mRNA encoding the enzyme in WT BMMs where a ~50% reduction in *Ch25h* mRNA was the maximum knockdown achieved (with siRNA 3 oligomer − here after referred to as *si*Ch25h) (Fig 3b). LDs were stained and measured and found to be significantly increased by ~3.5-fold in *si*Ch25h BMMs compared to control BMMs (Fig 3c; as seen in Fig 3d). ADRP staining shows that LDs in *si*Ch25h BMMs are dramatically larger than in control cells, as confirmed with quantification (Fig 3e). Thus the effect of reducing mRNA expression of *Ch25h* by approximately 50% was to dramatically increase lipid accumulation and LD size in WT BMMs. This is consistent with the increase in LDs in *sg/sg* cells where Ch25h is depleted.

25HC can be added exogenously to cells, and we sought to ‘rescue’ the *sg/sg* LD phenotype by adding back 25HC to these cells. Treatment of *sg/sg* BMMs with 25HC significantly decreased lipid accumulation into LD by approximately ~1.7-fold (Fig 3f). Taken together, the reduction of LDs after Ch25h knockdown in WT cells, and the reduction of over-accumulated LDs in *sg/sg* cells, provide evidence linking 25HC with LD homeostasis in macrophages. Specifically these results suggest that the loss of Ch25h in *sg/sg* macrophages at least partly explains their tendency to accumulate lipid.

Interestingly, a known effect of adding 25HC to normal cells is to increase LDs [17]. When we applied 25HC to WT BMMs it accordingly produced a dose-dependent increase in LD accumulation (S1a Fig). In further studies we treated *sg/sg* BMMS with supra-physiological levels of 25HC. The results show that, while at 100 nM, 25HC significantly decreased LDs in *sg/sg* BMMs by ~2-fold (similar to Fig 3f), and at higher doses it increased LDs, similar to WT cells (S1b Fig). Thus after correcting 25HC levels, and normalizing lipid homeostasis in *sg/sg* BMMs, excess 25HC then reverts to enhancing LDs and the cholesterol esterification for which it is known [17]. This suggests that RORα and 25HC are both working, perhaps in concert, to regulate lipid levels and LDs in macrophages. It prompted us to seek other components of this regulatory pathway that could affect LD homeostasis.

### Altered lipid handling by Ch25h and in *sg/sg* BMMs

**Fig 4:**
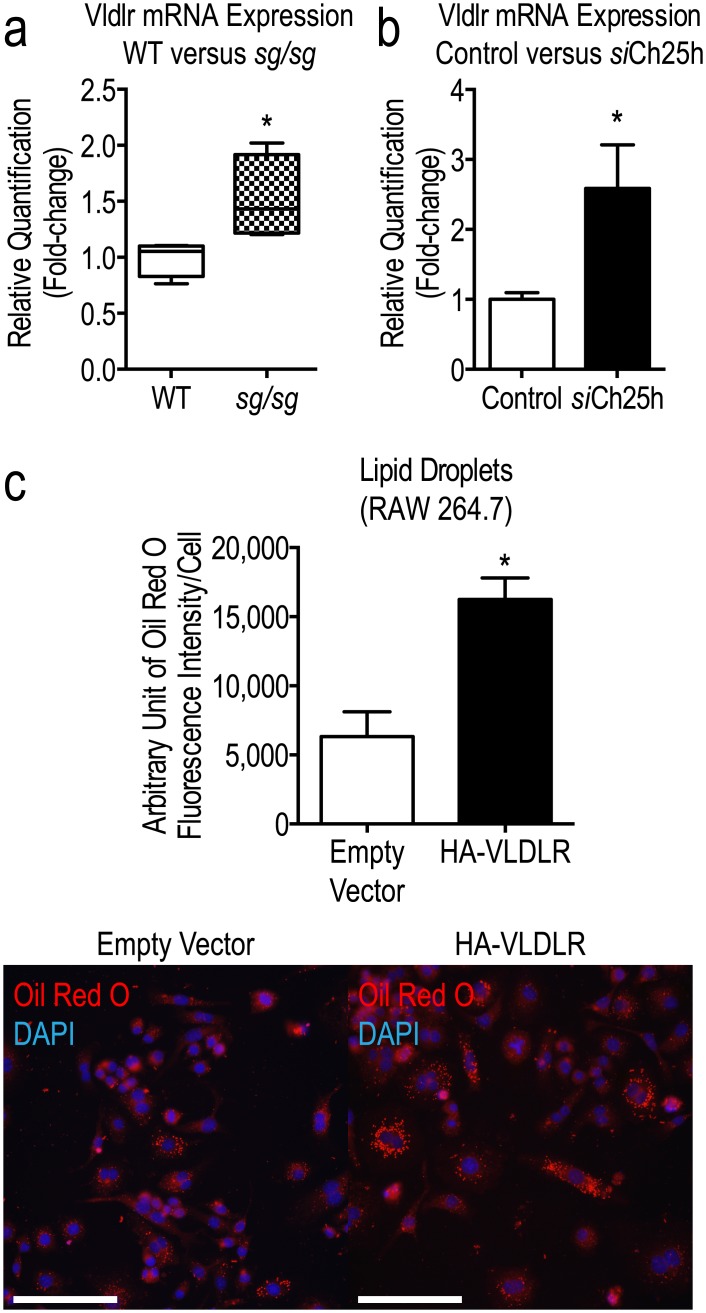
Increased *Vldlr* mRNA expression correlates with increased lipid uptake in *sg/sg* BMMs. (a) *Vldlr* mRNA expression from Lipoprotein Signaling and Cholesterol Metabolism RT^2^ Profiler PCR Array (Qiagen) expressed as the mean ± S.E.M. relative quantification (RQ) (fold-change) in *si*Ch25h cells (black bar) compared to control siRNA (set as 1.0; white bar). The Genorm software embedded in StatMiner determined *Actb*, *Gapdh*, and *Hsp90ab1* to be the three most stable and robust endogenous controls and all data were normalized to the median of their Ct values. The P-values were calculated using empirical Bayes statistics where *P<0.05. (b) Targeted qPCR analysis was performed using Taqman Gene Expression Assays for *Vldlr*. *Hprt1* was used as the endogenous control. Analysis was performed using RNA acquired from n = 4 littermate pairs of WT and *sg/sg* BMMs in triplicate experiments and represented as the mean ± S.E.M relative quantification (fold-change) with WT set as 1.0. Statistical analysis was performed using unpaired two-tailed Student’s t-test where *P<0.05; **P<0.01. (c) RAW264.7 cells were transiently transfected with either an empty HA-tagged pcDNA3.1 vector or a vector containing HA-VLDLR for 48 h. Cells were stained for LDs using Oil Red O and quantification of LDs is expressed as the mean ± S.E.M. fluorescence intensity (integrated density) of stained LDs normalized to number of cells analyzed (~350 cells per experiment, n = 3 independent experiments). Statistical analysis was performed using an unpaired two-tailed Student’s t-test where *P<0.05. Representative images of RAW264.7 cells transiently transfected with either an empty HA-tagged pcDNA3.1 vector or a vector containing HA-VLDLR for 48 h were captured using the Olympus upright wide-field epifluorescence microscope are shown in the bottom panel. Red denotes LDs (Oil Red O staining) and blue denotes nuclei (DAPI). Scale bars = 100 μm.

Preliminary data from microarray profiling of *sg/sg* BMMs performed previously [23] suggested that lipid uptake may be affected. Specifically, genes encoding cholesterol trafficking machinery and regulators were altered in the *sg/sg* mutant mice. mRNAs for peroxisome proliferator-activated receptor gamma (*Pparg*) and very low-density lipoprotein receptor (*Vldlr*) were up-regulated in *sg/sg* BMMs by ~1.32-fold and ~1.26-fold respectively (data not shown). We next profiled the mRNA expression of *Vldlr* and ~80 other genes known to participate in lipoprotein transport, signaling and cholesterol metabolism in *si*Ch25h macrophages. The analysis revealed changes that broadly reflect increased lipid uptake in these cells (Table 1, remaining list of non-significant probes are provided in S1 Table). For instance, *Vldlr* mRNA expression was significantly increased in *si*Ch25h-transfected BMMs compared to control cells (Table 1 and Fig 4a), consistent with the increased lipid accumulation phenotype observed in Fig 3. We also note that *Vldlr* mRNA expression is increased in *sg/sg* mice (Fig 4b). Finally, we can demonstrate VLDLR’s capacity for increasing LD size when the gene is overexpressed in RAW264.7 macrophages (Fig 4c). These results show that reduction of Ch25h is affecting lipid handling, establishing a link between 25HC and lipid storage potentially via VLDLR-mediated uptake pathways.

**Table 1:** Significant hits from cholesterol metabolism PCR array in *si*Ch25h cells versus control.

Gene Symbol	Relative Quantification (RQ)	Log_10_RQ	P-value	B-value	*t*-value	Ct Status	Significance
Ankra2	1.6464	0.2165	0.0450	-4.0362	-2.4902	Valid	P<0.05
Apoa1	0.0228	-1.6419	0.0046	-1.7226	4.2737	Target not detected	P<0.01
Lcat	1.6325	0.2129	0.0290	-3.5954	-2.8088	Valid	P<0.05
Lipe	1.7651	0.2468	0.0368	-3.8367	-2.6339	Valid	P<0.05
Vldlr	2.4081	0.3817	0.0164	-3.0192	-3.2350	Valid	P<0.05

‘Target not detected’ indicates undetermined Ct Status in target samples (*si*Ch25h).

## Discussion

Here we report for the first time that in the absence of Rorα, there is enhanced lipid accumulation in macrophages, producing the larger LDs that can be seen in BMMs from *sg/sg* mice. As active repositories of lipid, abnormal LDs reflect a concomitant imbalance in lipid trafficking and the abnormal lipid handling in macrophages now adds to the overall dysregulation of lipid storage and metabolism that characterize this mouse model, further evidencing a role for Rorα in obesity and inflammation. The findings reported here confirm the link we reported previously between loss of Rorα and decreased levels of the oxysterol 25HC [23]. Moreover, we now extend this pathway by showing increased lipid storage through an association of reduced RORα and 25HC in *sg/sg* BMMs.

The biology of 25HC is complex. It has potent and diverse effects on the governance of cellular sterol levels, in addition to functioning as a ligand for nuclear hormone receptors intimately associated with regulating lipid homeostasis (such as LXRs and RORs) [18, 21, 22]. 25HC can concomitantly suppress cholesterol biosynthesis and increase cholesterol esterification through interactions with other lipid homeostatic machinery [16, 17]. There have been no reports on the effects of 25HC deficiency on lipid handling to date and this may partly be due to the purported absence of an obvious metabolic phenotype in mice lacking Ch25h [15]. However, there is precedence for abnormal lipid storage in cells or systems deficient in oxysterol levels. Early studies on patients with a rare genetic disease characterized by the deficiency of an enzyme that produces a closely related oxysterol, 27-hydroxycholesterol, revealed that these patients develop premature atherosclerosis in spite of normocholesterolemia (cerebrotendinous xanthormatosis) [31]. The authors proposed that conversion of cholesterol to oxysterols in macrophages may facilitate cholesterol flux processes and provide protection in the presence of excess cholesterol. Loss of this protective mechanism may therefore dysregulate accumulation of cholesterol and contribute to premature atherosclerosis in these patients.

Certainly in the case of *sg/sg* BMMs, the deficiency in 25HC likely contributed to dysregulation of cholesterol sensing and related processes, resulting in increased lipid uptake and accumulation in the macrophages. However, the duality in the spectrum of 25HC’s effects on *sg/sg* BMMs (in reverting lipid storage defect when replenished with physiological levels of 25HC, and eventual increase in lipid storage at supraphysiological levels) also indicates that this is a complex relationship. Overall, our results suggest that macrophages are innately sensitive to exogenous 25HC treatment, producing different effects dependent on the baseline level of the oxysterol present within cells. It is apparent that 25HC in macrophages must be tightly regulated for optimal macrophage function, including for the efficient phagocytosis as a key innate immune response [23]. We propose that 25HC, working through RORα, is a major driver of lipid uptake and LD homeostasis predisposing cells to foam cell development and disease. 25HC is increasingly emerging with roles at the nexus of immune and metabolic signaling. Recent studies have implicated 25HC as a critical component of antiviral response and the effect was augmented in cells grown in a delipidized environment [32].

Both reduction of Ch25h and the *sg/sg* phenotype are characterized by altered expression of lipid handling regulators including VLDLR. An increase in VLDLR expression supports the increased lipid accumulation and uptake phenotypes presented in the macrophages. Expression of VLDLR is markedly increased during atherosclerotic lesion development [33, 34], correlating with the atherosclerotic tendency of *sg/sg* mice. We confirmed the correlation with increased LDs as a result of increased VLDLR expression in a macrophage cell line, reflecting the results obtained in another study on cardiomyocyte cells [35]. Thus the effect of up-regulated VLDLR expression is to increase lipid uptake and storage, highlighting this as a possible player in the lipid handling pathways modulated by 25HC and RORα. Further studies would be required to confirm the nature of the link between 25HC, RORα and VLDLR in this context.

Thus, our findings implicate a new role for RORα and 25HC in the governance of lipid storage in macrophages though VLDLR, extending the importance of regulating optimal 25HC levels and nuclear hormone receptor signaling for macrophage lipid metabolism. This in turn impacts on chronic diseases, particularly those influenced heavily by our modern day life-styles, such as diabetes and obesity.

## Supporting Information

S1 Fig Assessment of LDs in WT and *sg/sg* BMMs treated with 25HC. (a) Representative images of LDs in WT BMMs treated with vehicle control (0.01% DMSO), low levels (*top*, 0.025 μM to 0.1 μM) or high levels of 25HC (*bottom*, 0.1 μM and 2 μM 25HC) for 4 h. (b) Representative images of LDs in *sg/sg* BMMs treated with vehicle control (0.01% DMSO) or high levels of 25HC (*bottom*, 0.1 μM and 2 μM 25HC) for 4 h. Blue denotes nucleus (DAPI) labeling and red denotes LD (Oil Red O) labeling. All images were captured using the were captured using the Olympus upright wide-field epifluorescence microscope. Quantification of LDs is expressed as the mean ± S.E.M. fluorescence intensity (integrated density) of stained LDs normalized to number of cells analyzed (low 25HC, n = ~100 cells per mouse per treatment; high 25HC, n = ~300 cells per mouse per treatment). Statistical analyses were performed using one-way ANOVAs with Bonferroni’s post test (n = 3–4 biological replicates), comparing the amount of LDs in each treatment to the vehicle control where *P<0.05; **P<0.01; ***P<0.001; n.s. denotes non-significant. Scale bars = 50 μm.
(EPS)

S1 Table Gene expression changes and statistics for all probes on the Lipoprotein Signaling & Cholesterol Metabolism PCR Array (PAMM-080Z) platform in *si*Ch25h BMMs relative to control. No detection indicates undetermined Ct Status in both control and target samples.
(DOCX)

## Abbreviations

25HC: 25-Hydroxycholesterol
Abc: ATP-binding cassette
acLDL: Acetylated LDL
ADRP: Adipophilin
BCA: Bicinchoninic acid
BMM: Bone marrow-derived macrophages
Ch25h: Cholesterol 25-hydroxylase
DMSO: Dimethyl sulfoxide
ECL: Enhanced chemiluminescence
HDL: High density lipoprotein
HRP: Horseradish peroxidase
LD: Lipid droplet
LDL: Low-density lipoprotein
LXR: Liver X receptor
M-CSF-1: Macrophage-colony-stimulating-factor-1
mRNA: Messenger RNA
n.a.: Numerical aperture
PBS: Phosphate buffered saline
PCR: Polymerase chain reaction
PFA: Paraformaldehyde
PVDF: Polyvinyldene fluoride
qPCR: Quantitiative real-time PCR
RNA: Ribonucleic acid
RORα: Retinoid-related orphan receptor alpha
RQ: Relative quantification
SDS-PAGE: Sodium dodecyl sulfate-polyacrylamide gel electrophoresis
sg/sg: *Staggerer*
*si*Ch25h: Ch25h-knockdown cells
siRNA: Small interfering RNA
VLDLR: Very low-density lipoprotein receptor
WT: Wild-type
