# Supplementary material for: RORα and 25-Hydroxycholesterol Crosstalk Regulates Lipid Droplet Homeostasis in Macrophages
Source: PLoS One. 2016 Jan 26;11(1):e0147179. doi: 10.1371/journal.pone.0147179 (PMC4727927; doi:10.1371/journal.pone.0147179)
Supplement: S1 Table — No detection indicates undetermined Ct Status in both control and target samples. (DOCX) [file pone.0147179.s002.docx]

**S1 Table.**

| **Gene Symbol** | **Relative Quantification (RQ)** | **Log_10_RQ** | **P-value** | **B-value** | ***t*-value** | **Ct Status** | **Significance** |
| --- | --- | --- | --- | --- | --- | --- | --- |
| Ankra2 | 1.6464 | 0.2165 | 0.0450 | -4.0362 | -2.4902 | Valid | Significant |
| Apoa1 | 0.0228 | -1.6419 | 0.0046 | -1.7226 | 4.2737 | Target not detected | Significant |
| Lcat | 1.6325 | 0.2129 | 0.0290 | -3.5954 | -2.8088 | Valid | Significant |
| Lipe | 1.7651 | 0.2468 | 0.0368 | -3.8367 | -2.6339 | Valid | Significant |
| Vldlr | 2.4081 | 0.3817 | 0.0164 | -3.0192 | -3.2350 | Valid | Significant |
| Abca1 | 2.0776 | 0.3176 | 0.1182 | -4.9760 | -1.8061 | Valid | Non Significant |
| Abca2 | 1.2414 | 0.0939 | 0.3807 | -5.9859 | -0.9419 | Valid | Non Significant |
| Abcg1 | 2.1591 | 0.3343 | 0.0927 | -4.7448 | -1.9777 | Valid | Non Significant |
| Acaa2 | 1.3901 | 0.1430 | 0.2891 | -5.7703 | -1.1566 | Valid | Non Significant |
| Akr1d1 | 4.4645 | 0.6498 | 0.3219 | -5.8568 | -1.0740 | No detection | Non Significant |
| Angptl3 | 1.8596 | 0.2694 | 0.0983 | -4.8008 | -1.9365 | Valid | Non Significant |
| Apoa2 | 1.4287 | 0.1550 | 0.1333 | -5.0884 | -1.7211 | Valid | Non Significant |
| Apoa4 | 1.3909 | 0.1433 | 0.1890 | -5.4064 | -1.4713 | No detection | Non Significant |
| Apob | 1.3909 | 0.1433 | 0.1890 | -5.4064 | -1.4713 | No detection | Non Significant |
| Apoc3 | 1.3909 | 0.1433 | 0.1890 | -5.4064 | -1.4713 | No detection | Non Significant |
| Apod | 0.3575 | -0.4467 | 0.6916 | -6.3580 | 0.4152 | Valid | Non Significant |
| Apoe | 1.4867 | 0.1722 | 0.1047 | -4.8612 | -1.8918 | Valid | Non Significant |
| Apof | 1.1398 | 0.0568 | 0.8353 | -6.4276 | -0.2167 | Valid | Non Significant |
| Apol8 | 1.9494 | 0.2899 | 0.7729 | -6.4032 | -0.3012 | Valid | Non Significant |
| Cdh13 | 0.0681 | -1.1669 | 0.1553 | -5.2294 | 1.6123 | Valid | Non Significant |
| Cel | 0.3554 | -0.4493 | 0.6932 | -6.3590 | 0.4130 | Valid | Non Significant |
| Cela3b | 6.0618 | 0.7826 | 0.2530 | -5.6597 | -1.2569 | No detection | Non Significant |
| Cnbp | 1.3831 | 0.1409 | 0.1842 | -5.3836 | -1.4898 | Valid | Non Significant |
| Colec12 | 1.2414 | 0.0939 | 0.3749 | -5.9744 | -0.9542 | Valid | Non Significant |
| Crp | 1.3909 | 0.1433 | 0.1890 | -5.4064 | -1.4713 | No detection | Non Significant |
| Cxcl16 | 1.4380 | 0.1578 | 0.2262 | -5.5641 | -1.3400 | Valid | Non Significant |
| Cyb5r3 | 0.8037 | -0.0949 | 0.2693 | -5.7117 | 1.2103 | Valid | Non Significant |
| Cyp11a1 | 1.3909 | 0.1433 | 0.1890 | -5.4064 | -1.4713 | No detection | Non Significant |
| Cyp39a1 | 2.2706 | 0.3561 | 0.0793 | -4.5945 | -2.0875 | Valid | Non Significant |
| Cyp46a1 | 0.5638 | -0.2489 | 0.7862 | -6.4091 | 0.2830 | Valid | Non Significant |
| Cyp51 | 1.2832 | 0.1083 | 0.5044 | -6.1822 | -0.7075 | Valid | Non Significant |
| Cyp7a1 | 1.3909 | 0.1433 | 0.1890 | -5.4064 | -1.4713 | No detection | Non Significant |
| Cyp7b1 | 5.0868 | 0.7064 | 0.2556 | -5.6683 | -1.2493 | No detection | Non Significant |
| Dhcr24 | 0.9750 | -0.0110 | 0.9354 | -6.4500 | 0.0843 | Valid | Non Significant |
| Dhcr7 | 1.3527 | 0.1312 | 0.2568 | -5.6721 | -1.2459 | Valid | Non Significant |
| Ebp | 1.3151 | 0.1190 | 0.2318 | -5.5851 | -1.3220 | Valid | Non Significant |
| Fdft1 | 1.2302 | 0.0900 | 0.3970 | -6.0169 | -0.9081 | Valid | Non Significant |
| Fdps | 1.1607 | 0.0647 | 0.5488 | -6.2348 | -0.6330 | Valid | Non Significant |
| Hdlbp | 1.0757 | 0.0317 | 0.7539 | -6.3940 | -0.3274 | Valid | Non Significant |
| Hmgcr | 1.0401 | 0.0171 | 0.8820 | -6.4406 | -0.1545 | Valid | Non Significant |
| Hmgcs1 | 1.4872 | 0.1724 | 0.3006 | -5.8019 | -1.1268 | Valid | Non Significant |
| Hmgcs2 | 5.8129 | 0.7644 | 0.3300 | -5.8763 | -1.0548 | No detection | Non Significant |
| Idi1 | 1.3270 | 0.1229 | 0.4469 | -6.1014 | -0.8106 | Valid | Non Significant |
| Idi2 | 0.3928 | -0.4058 | 0.6683 | -6.3420 | 0.4491 | Valid | Non Significant |
| Insig1 | 1.3392 | 0.1268 | 0.2795 | -5.7427 | -1.1821 | Valid | Non Significant |
| Insig2 | 1.2502 | 0.0970 | 0.3030 | -5.8084 | -1.1207 | Valid | Non Significant |
| Ldlr | 0.9940 | -0.0026 | 0.9831 | -6.4538 | 0.0221 | Valid | Non Significant |
| Ldlrap1 | 1.2302 | 0.0900 | 0.2434 | -5.6269 | -1.2857 | Valid | Non Significant |
| Lep | 0.6679 | -0.1753 | 0.5975 | -6.2839 | 0.5556 | No detection | Non Significant |
| Lrp10 | 1.3486 | 0.1299 | 0.2527 | -5.6586 | -1.2579 | Valid | Non Significant |
| Lrp12 | 1.5614 | 0.1935 | 0.3151 | -5.8397 | -1.0907 | Valid | Non Significant |
| Lrp1b | 0.7008 | -0.1544 | 0.5123 | -6.1923 | 0.6938 | No detection | Non Significant |
| Lrp6 | 1.5176 | 0.1811 | 0.1102 | -4.9099 | -1.8556 | Valid | Non Significant |
| Lrpap1 | 1.3744 | 0.1381 | 0.1544 | -5.2241 | -1.6164 | Valid | Non Significant |
| Mbtps1 | 1.2992 | 0.1137 | 0.2680 | -5.7080 | -1.2137 | Valid | Non Significant |
| Mvd | 1.3102 | 0.1173 | 0.2812 | -5.7475 | -1.1776 | Valid | Non Significant |
| Mvk | 1.0157 | 0.0067 | 0.9397 | -6.4506 | -0.0787 | Valid | Non Significant |
| Npc1l1 | 5.3949 | 0.7320 | 0.4875 | -6.1602 | -0.7368 | Valid | Non Significant |
| Nr0b2 | 0.9452 | -0.0245 | 0.7344 | -6.3838 | 0.3546 | Valid | Non Significant |
| Nr1h4 | 1.0731 | 0.0306 | 0.9561 | -6.4522 | -0.0572 | Target not detected | Non Significant |
| Nsdhl | 1.1656 | 0.0666 | 0.2776 | -5.7371 | -1.1872 | Valid | Non Significant |
| Olr1 | 0.0761 | -1.1188 | 0.2163 | -5.5253 | 1.3728 | Valid | Non Significant |
| Osbpl1a | 1.2396 | 0.0933 | 0.4282 | -6.0715 | -0.8462 | Valid | Non Significant |
| Osbpl5 | 1.2953 | 0.1124 | 0.4295 | -6.0736 | -0.8436 | Valid | Non Significant |
| Pcsk9 | 1.6700 | 0.2227 | 0.0590 | -4.3050 | -2.2968 | Valid | Non Significant |
| Pmvk | 1.3311 | 0.1242 | 0.1756 | -5.3408 | -1.5242 | Valid | Non Significant |
| Ppard | 1.3945 | 0.1444 | 0.1011 | -4.8279 | -1.9165 | Valid | Non Significant |
| Prkaa1 | 1.5335 | 0.1857 | 0.0510 | -4.1613 | -2.4002 | Valid | Non Significant |
| Prkaa2 | 1.3104 | 0.1174 | 0.3400 | -5.8996 | -1.0316 | Valid | Non Significant |
| Prkag2 | 1.1174 | 0.0482 | 0.5203 | -6.2020 | -0.6803 | Valid | Non Significant |
| Scap | 1.1261 | 0.0516 | 0.4624 | -6.1247 | -0.7820 | Valid | Non Significant |
| Scarf1 | 1.0983 | 0.0407 | 0.5677 | -6.2549 | -0.6025 | Valid | Non Significant |
| Snx17 | 0.9653 | -0.0154 | 0.7826 | -6.4076 | 0.2878 | Valid | Non Significant |
| Soat1 | 1.1304 | 0.0532 | 0.3399 | -5.8994 | -1.0317 | Valid | Non Significant |
| Soat2 | 1.0614 | 0.0259 | 0.7755 | -6.4044 | -0.2976 | Valid | Non Significant |
| Sorl1 | 1.0231 | 0.0099 | 0.9172 | -6.4475 | -0.1082 | Valid | Non Significant |
| Srebf1 | 1.0058 | 0.0025 | 0.9751 | -6.4535 | -0.0325 | Valid | Non Significant |
| Srebf2 | 1.0600 | 0.0253 | 0.6869 | -6.3549 | -0.4221 | Valid | Non Significant |
| Stab1 | 1.1137 | 0.0468 | 0.6431 | -6.3230 | -0.4863 | Valid | Non Significant |
| Stab2 | 1.2452 | 0.0952 | 0.3167 | -5.8440 | -1.0865 | Valid | Non Significant |
| Stard3 | 1.1861 | 0.0741 | 0.3242 | -5.8623 | -1.0686 | Valid | Non Significant |
| Tm7sf2 | 0.8966 | -0.0474 | 0.6382 | -6.3191 | 0.4936 | Valid | Non Significant |
| Trerf1 | 1.2351 | 0.0917 | 0.2103 | -5.5010 | -1.3933 | Valid | Non Significant |
